# Characteristics of nursing interventions that improve the quality of life of people with chronic diseases. A systematic review with meta-analysis

**DOI:** 10.1371/journal.pone.0218903

**Published:** 2019-06-24

**Authors:** Francisco José Amo-Setién, Rebeca Abajas-Bustillo, Blanca Torres-Manrique, Roberto Martín-Melón, Carmen Sarabia-Cobo, Jesús Molina-Mula, Carmen Ortego-Mate

**Affiliations:** 1 Faculty of Nursing, University of Cantabria, IDIVAL Nursing Group, Santander, Spain; 2 Biosciences Library, University of Cantabria, Santander, Spain; 3 Nursing and Physiotherapy Department, University of Illes Balears, Palma, Spain; University of Mississippi Medical Center, UNITED STATES

## Abstract

**Purpose:**

The objective of this systematic review was to determine the characteristics of the interventions conducted by nurses that attempt to improve the health related quality of life (HRQoL) of people over 18 years of age with chronic diseases.

**Methods:**

This systematic review with meta-analysis summarizes 24 studies, conducted in 10 countries, that evaluated HRQoL through the Short-Form Health Survey (SF). Five databases were accessed to find the available studies from December 31^st^, 2000 to May 22^sd^, 2017. Selected studies were coded according to the characteristics of the sample and the intervention. A model of random effects was adopted for the overall estimation and to explain the heterogeneity.

**Results:**

Twenty-four studies were included in the systematic review and meta-analysis providing a sample of 4324 chronic patients aged 63.4 years. Among the 8 subscales and two summary measures that comprise the SF-36, only an overall significant effect size (ES) index was found in the Mental Health Component summary score (ES = 0.14; 95% CI:0.03 − 0.26; I^2^ = 44.6, p = 0.042) and the Mental Health subscale. This improvement on HRQoL was associated to interventions on “Case Management” and “Treatments and Procedures”, which were based on a theory, were of shorter duration, and had a follow-up period.

**Conclusions:**

Interventions targeting people with chronic diseases resulted in a slight increase in the HRQoL that was not always significant, which suggests that there is a need for their continuous improvement.

## Introduction

In the year 2018, the World Health Organization (WHO) estimated that 71% of the deaths worldwide were due to chronic diseases, and this situation was expected to worsen [1]. Among chronic diseases, also known as Non Communicable Diseases (NCDs), the heart diseases, cerebrovascular accidents, cancer, chronic respiratory diseases and diabetes, were highlighted [2]. The fight against NCDs has become one of the main challenges for health systems [3]. They affect not only physical health, but also a person’s psychological state, their level of autonomy and the skills needed to perform daily tasks at work and in society [4–7]. These diseases, besides being permanent, can be associated to functional limitations in different dimensions, and this is why treatments are frequently geared towards the improvement of overall health [3,8,9].

According to the WHO, nurses and midwives make up almost 50% of the workforce of health professionals, and their figure must be strengthened in order to support universal health coverage and thus improving the health of the different populations [10]. Due to their close contact with patients and their families, nurses perform interventions focused on the population with NCDs. In these interventions, one of the parameters that is frequently assessed and attempted to improve is health-related quality of life (HRQoL). According to Shumaker et al. [11] it can be understood as “*people's subjective evaluations of the influences of their current health status*, *health care*, *and health-promoting activities on their ability to achieve and maintain a level of overall functioning that allows them to pursue valued life goals*, *and that is reflected in their general well-being*”.

The main reason for using quality of life measurements in clinical practice is that it allows treatments to be patient- rather than disease-centered [3,12]. Given their ability to focus on the real needs perceived by the population, the determination of HRQoL is considered a highly discriminative tool in the planning of health policies or in the distribution of resources [13–15].

There are many questionnaires that can be used to evaluate HRQoL, and they differ in their structure (profiles vs indices), as well as in the area of application (generic vs specific) [12]. In general, these questionnaires encompass questions that refer to the severity or intensity of symptoms, functional impairment, emotional disorders and the perception of well-being [3,12].

The Short Form-36 Health Survey (SF-36) is probably the most commonly used instrument for measuring HRQoL [16]. It is a general scale that provides a profile of health status, and is applicable to both chronic patients and the general population [16,17]. Many studies have defined it as having good reliability and construct validity for populations of people with chronic diseases [18–21].

Due to the importance that patients with chronic diseases attach to the improvement or at least the maintenance of HRQoL, the objective of this systematic review was to determine the characteristics of the interventions conducted by nurses aimed at improving the HRQoL of people over 18 years of age with chronic diseases. Any activity outside the usual care programmes aimed at improving the quality of life of chronically ill patients and conducted by nurses has been considered an intervention. Usual care was commonly employed in the control group.

## Materials and methods

### Selection criteria and search strategy

In order to select the studies to be used in the systematic review, a search in 5 electronic databases was conducted by an expert on bibliographic search methodology (RM). The databases consulted were: Medline (Pubmed), Scopus, Web of Science, CINAHL and Cochrane.

To conduct the search, boolean operators “AND” and “OR” were used to combine the search terms, which in some cases were truncated to generate the maximum number of results:

(“chronic disease*” OR “chronically ill” OR “chronically disease*” OR “chronic illness*” OR “chronically critically ill” OR CCI OR “Chronic patient*” OR “noncommunicable disease*” OR “non communicable disease* OR NCD OR comorbidit* OR multimorbidit* OR “complex patient*”) AND (“quality of life” OR “life quality” OR QOL OR HRQOL OR HRQL OR “short-form” OR SF36 OR SF12) AND nurs* AND (“randomized controlled trial” OR RCT OR “clinical trial”). Limits used: Adults, English, Spanish, Time period = 2000–2017.

Some of the above terms were recognized and used as MeSH terms by the Pubmed search engine: “Chronic Disease”, “Multiple Chronic Conditions”, “Noncommunicable Diseases”, “Quality of Life”, “Nurse” and “Nursing”.

Likewise, a secondary manual exploration of references cited in the studies selected was performed in order to find primary studies as well as systematic reviews and meta-analysis, related to the topic of interest and not identified in the primary search. Peer review of titles in the first place and abstracts in the second place served to determine whether the results met the inclusion criteria before reading the full-texts. The search was conducted in 2017 and restricted to adults (>18 years old), studies written in Spanish or English, and published from December 31st, 2000 to May 22sd, 2017.

The inclusion criteria of primary studies in the systematic review were the following:

- Randomized controlled trial.
- Participation of at least one nurse in the intervention.
- The intervention was performed on chronic patients >18 year of age.
- The study had to include at least two groups, one of them being the control group.
- The study provided mean values, or the mean change between pre and post values of the intervention and control groups, standard deviations and sample size post intervention of both groups in any of the two summary measures or 8 subscales of the SF-36. Studies using SF-12 were also included, since this version provides the result of the two summary measures.
- The study had to be written in Spanish or English.

The results on HRQoL from the Short Form Health Survey of each study were recorded. This questionnaire is composed of 36 questions (items) that evaluate both positive and negative health states. These 36 items are distributed among 8 subscales: Physical Functioning (PF), Role-Physical (RP), Bodily Pain (BP), General Health (GH), Vitality (VT), Social Functioning (SF), Role-Emotional (RE) and Mental Health (MH). At the same time, these 8 subscales can be included within two summary measures: Physical Component Summary (PCS) and Mental Health Component Summary (MCS).

To perform the systematic review, a protocol that included a coding manual, the coding or data extraction sheets and the work procedure was created (S1 File).

Each selected study was independently coded by two coders (F.J.A-S and R.A-B), who extracted the data using a data collection sheet and the coding manual created for this purpose. Disagreements were solved by a third-party expert (C.O-M). The design of both instruments was based on the variables coded in published meta-analyses related to interventions and previous experience.

Two reviewers independently assessed the methodological quality of the RCTs according to the Cochrane Manual 5.1.0 guidelines, using 7 items, each of which had three response options: “Low”, “High” and “Not clear” [22].

Once the studies were coded, the intercoder agreement was estimated, resulting in a mean agreement reached for both categorical and continuous variables. The disagreements were solved through deliberation between coders, and when necessary, a third reviewer's criterion was used.

### Characteristics of the interventions

The following intervention characteristics were collected:

- Theory: contextual framework used as a foundation for developing the intervention. The presence or absence of theory was recorded, as well as its name.
- Incentive: financial or in-kind remuneration offered to patients for their participation
- Multidisciplinarity: whether the intervention was led by a multidisciplinary team or exclusively by nurses.
- Prior training: the specific pre-training that staff received to perform the intervention.
- Type of intervention: each intervention was classified in one or more of the four categories of the Omaha System intervention classification: “Teaching, Guidance, and Counseling”, “Treatments and Procedures”, “Case Management”, “Surveillance”. A description of each can be found on the official Omaha website [23].
- Context: the location where the intervention was performed, either in a clinical context, in a private home, or both.
- Type of contact: if the contact with the subject receiving the intervention was direct (in person), indirect (at a distance, via telephone, e-mail …) or received both types of contact.
- Duration of the intervention: the time dedicated to carrying out the complete intervention, in weeks, excluding the follow-up period.
- Follow-up period: the time dedicated to the evaluation of the outcomes in the patient after the intervention is finished, to check the evolution and persistence of the effects. The duration of the follow-up was not taken into account, only if it was present or not. The studies that did not have follow up period evaluated the results "post" only at the end of the intervention.
- Sessions: the number of sessions of the intervention.
- Time per session: duration in minutes of each of the sessions in which the intervention took place.

Also, a detailed and qualitative description of the interventions can be found in S2 File.

### Statistical analyses

The standard differences in means proposed by Hedges in each study were weighted by the inverse of their variance in order to obtain the pooled index of the magnitude of the effect, following random-effects assumptions as the results are more robust than fixed-effects assumptions.

Bias in the effect sizes distribution and sensitivity analysis were conducted. More details on statistical analysis are reported in S3 File.

## Results

**Fig 1:**
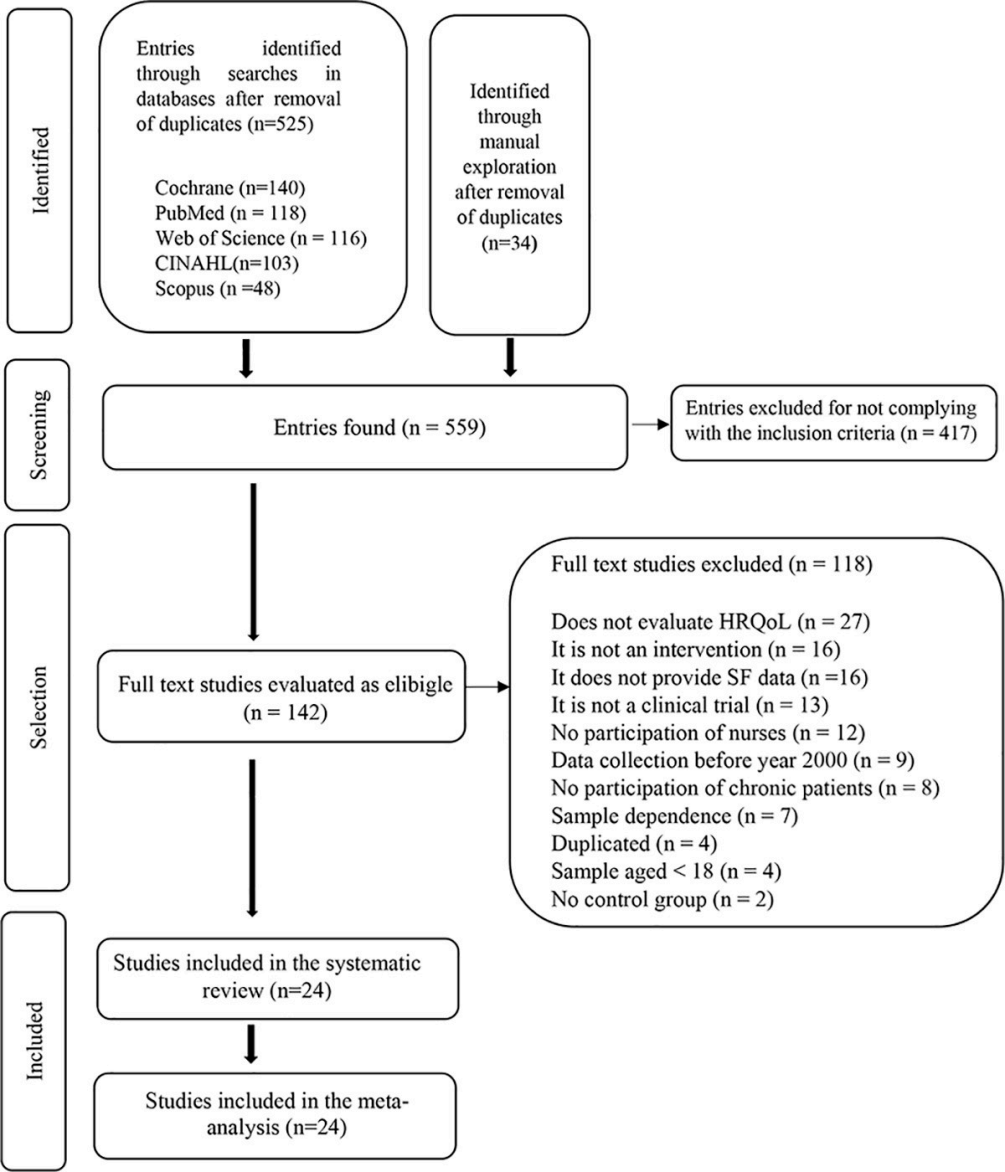
Flow diagram. Selection process.

The exhaustive search began on May 5th, 2017, and ended on January 9th, 2018, and resulted in 559 studies, of which 24 were included in the systematic review and meta-analysis [24–47]. Fig 1 shows the flow diagram of the review process, in agreement with PRISMA guidelines [48].

From these 24 studies, 30 intervention groups and 29 control groups were extracted, as four studies provided two intervention groups, one study contributed with three intervention groups, and nineteen studies provided an intervention group and a control group. Tables 1 and 2 show the characteristics of the 24 studies included in this systematic review and the characteristics of their interventions.

**Table 1:** Description of studies included in the systematic review.

*Main author (publication year)*	*Data collection year*	*Funding*	*Country*^*a*^	*Intervention (n)*	*Control (n)*	*Average age*	*Chronic disease*^*b*^
Alexander and Wagner (2012)	-	No	USA	9	16	70.5	COPD
Angermann et al. (2012)	2004	Yes	Germany	352	363	68.6	Heart failure
Arvidsson et al. (2012)	2009	Yes	Sweden	38	124	55.8	Rheumatic disease
Berkhof et al. (2015)	2003	Yes	The Netherlands	52	49	68.0	COPD
Brodie et al. (2008)	2002	No	UK	42	18	77.0	Heart failure
Chow and Wong (2010)	2005	Yes	China	43	42	57.0	Kidney failure
Chow and Wong (2014)	2010	Yes	China	183	98	76.0	Chronic diseases
Coultas et al. (2005)	2000	Yes	USA	49	51	68.6	COPD
Donesky-Cuenco et al. (2009)	2004	Yes	USA	14	15	70.0	COPD
Friedberg et al. (2013)	2009	Yes	USA	37	36	43.1	Chronic fatigue
Gellis et al. (2014)	2010	Yes	USA	57	58	79.2	Multiple morbidities
Gensichen et al. (2009)	2005	Yes	Germany	267	288	51.1	Depression
Hendriks et al. (2014)	-	Yes	The Netherlands	286	248	66.7	Atrial fibrillation
Houweling et al. (2011)	-	Yes	The Netherlands	102	104	68.3	Diabetes
Jason et al. (2007)	-	Yes	USA	86	28		Chronic fatigue
Markle-Reid et al. (2006)	2001	Yes	Canada	120	122		Chronic diseases
McCorkle et al. (2009)	2003	Yes	USA	63	60	60.3	Gynecologic cancer
Peters-Klimm et al. (2010)	2006	Yes	Germany	97	100	69.7	Heart failure
Shearer et al. (2007)	2001	Yes	USA	42	45	76.0	Heart failure
Sorensen and Frich (2008)	2000	Yes	Denmark	52	49	52.3	Chronic pain
Tsai et al. (2015)	2012	No	Taiwan	32	25	63.0	Chronic disease
Tsay et al. (2005)	-	Yes	Taiwan	30	27	50.7	Kidney disease
Tummers et al. (2012)	-	Yes	The Netherlands	62	61	36.4	Chronic fatigue
Walters et al. (2013)	2008	Yes	Australia	90	92	67.8	COPD

^a^USA: United States of America; UK: United Kingdom

^b^COPD: Chronic obstructive pulmonary disease

**Table 2:** Characteristics of the interventions and results on SF-36.

*Author*	*Theory*	*QoL Scale*	*Duration* *(weeks)*	*Sessions*	*Type of* *intervention*	*Multidisciplinarity*	*Training*	*Follow-up*	*Results on Quality of Life (SF-36)*									
									*PCS*	*MCS*	*PF*	*RP*	*BP*	*GH*	*VT*	*SF*	*RE*	*MH*
Alexander and Wagner (2012)	No	SF-36	10	16	2	Yes	No	No			NS	NS	NS	NS	NS	NS	NS	NS
Angermann et al. (2012)	No	SF-36	26	9	4	No	Yes	No	+	NS	+							
Arvidsson et al. 2012	No	SF-36	52	10	1	No	No	Yes			NS	NS	NS	NS	NS	NS	NS	NS
Berkhof et al. (2015)	No	SF-36	26	13	3	Yes	No	No			NS	NS	NS	NS	NS	NS	NS	NS
Brodie et al. (2008)	No	SF-36	21	8	2	Yes	Yes	No			NS	+	NS	NS	NS	+	NS	NS
Chow and Wong (2010)	Yes	SF-36	6	6	4	No	Yes	No			NS	NS	NS	NS	NS	+	NS	NS
Chow and Wong (2014)	Yes	SF-36	4	4	4	No	No	Yes	+	NS	+	+	NS	NS	+	+	NS	+
Coultas et al. (2005)	No	SF-36	26	6	3	No	Yes	Yes			NS	NS	NS	NS	NS	NS	NS	NS
Donesky-Cuenco et al. (2009)	No	SF-36	12	24	2	Yes	No	No	NS	NS								
Friedberg et al. (2013)	No	SF-36	52	2	4	No	Yes	No			NS							
Gellis et al. (2014)	No	SF-12	13		4	No	Yes	Yes	NS	+								
Gensichen et al. (2009)	No	SF-36	52	19	4	No	Yes	No	NS	NS								
Hendriks et al. (2014)	No	SF-36	56	4	3	No	No	No			NS	NS	+	NS	NS	NS	+	NS
Houweling et al. (2011)	No	SF-36	60		3	Yes	Yes	No	–	NS	NS	NS	NS	NS	NS	NS	NS	NS
Jason et al. (2007)	No	SF-36	26	13	2	No	No	Yes			+							
Markle-Reid et al. (2006)	Yes	SF-36	26		3	No	No	No	NS	+	NS	NS	NS	NS	NS	NS	+	+
McCorkle et al. (2009)	No	SF-12	26	18	1	No	No	No	+	+								
Peters-Klimm et al. (2010)	No	SF-36	52	7	4	Yes	Yes	No	NS	NS	NS	NS	NS	NS	+	NS	NS	NS
Shearer et al. (2007)	Yes	SF-36	12	6	1	No	No	No	NS	NS								
Sorensen and Frich (2008)	No	SF-36	104	7	1	No	No	No			NS	NS	NS	NS	NS	NS	NS	NS
Tsai et al. (2015)	No	SF-36	4	8	2	No	Yes	No	NS	+	NS	NS	NS	NS	NS	NS	+	NS
Tsay et al. (2005)	Yes	SF-36	8	8	2	No	Yes	Yes	+	+								
Tummers et al. (2012)	No	SF-36	20		2	No	Yes	No			NS					NS		
Walters et al. (2013)	No	SF-36	52		1	No	Yes	No	NS	NS	NS	NS	NS	NS	NS	NS	NS	NS

QoL: Quality of Life; Type of intervention (OMAHA classification system): 1. Teaching, Guidance, and Counseling/ 2. Treatments and Procedures/ 3. Case Management/ 4. Surveillance. PCS: Physical Component Summary; MCS: Mental Health Component Summary; PF: Physical Functioning; RP: Role-Physical; BP: Bodily Pain; GH: General Health; VT: Vitality; SF: Social Functioning; RE: Role-Emotional; MH: Mental Health. NS: Non-significant (p ≥ 0.05). “+”: Significant and positive result; “–“: Significant and negative result.

Of these studies, 87.5% (n = 21) were funded, 66.7% (n = 8) were multi-centered, 41.7% (n = 10) were conducted in Western Europe, 37.5% (n = 9) in North America, 16.7% (n = 4) in Asia, and 4.2% (n = 1) in Oceania.

The 24 studies provided a sample of 4324 chronic patients (2205 from the intervention group and 2119 from the control group), with a mean age of 63.4 (SD: 11.3, 36–79 years of age).

The duration of the interventions ranged between 4–104 weeks, with a mean of 9.9 sessions and 44.3 minutes/session (SD = 31.4). In 79.0% (n = 19) of the studies, interventions were not based on a theory.

The intervention was exclusively performed by nurses in 75.0% (n = 18) of the studies, and in the rest of them, along with the nurse, other health professionals, such as pharmacists, doctor, other health worker, psychologists or others, took part.

To perform the intervention, 45.8% (n = 11) of the professionals did not receive specific training, and in 70.0% (n = 17) there was no follow-up period.

The most common type of intervention was “Case Management” [37.5% (n = 9)], followed by Treatments and Procedures [29.2% (n = 7)] and “Teaching, Guidance and Counselling” [20.8% (n = 5)].

Bias was evaluated following the Cochrane guidelines [22]. Of the seven items that assess the bias, the most monitored and best profiled bias was the generation of randomization, classified as "low" in 58.3% (n = 14) of the studies. For the rest of the items, the most frequent bias classification was "unclear" (Table 3).

**Table 3:** Risk of bias summary: Review authors' judgements about each risk of bias item for each included study.

*Main author*	*Bias*. *Author's judgement*^*a*^						
	*Random sequence generation (selection bias)*	*Allocation concealment (selection bias)*	*Blinding of participants and personnel (performance bias)*	*Blinding of outcome assessment (detection bias)*	*Incomplete outcome data (attrition bias)*	*Selective reporting (reporting bias)*	*Other bias*
Alexander and Wagner (2012)	3	3	1	3	3	3	3
Angermann et al. (2012)	2	2	1	2	2	2	2
Arvidsson et al. 2012	3	2	1	3	3	3	3
Berkhof et al. (2015)	2	3	1	2	2	2	3
Brodie et al. (2008)	2	2	1	3	1	1	3
Chow and Wong (2010)	2	3	1	3	3	3	3
Chow and Wong (2014)	2	2	1	2	3	2	3
Coultas et al. (2005)	2	3	1	2	3	3	3
Donesky-Cuenco et al. (2009)	3	3	1	1	2	1	3
Friedberg et al. (2013)	2	3	1	2	2	3	2
Gellis et al. (2014)	2	3	1	2	2	3	3
Gensichen et al. (2009)	3	3	1	1	2	2	2
Hendriks et al. (2014)	3	3	1	3	2	3	3
Houweling et al. (2011)	3	2	1	1	3	3	3
Jason et al. (2007)	2	3	1	3	3	3	3
Markle-Reid et al. (2006)	2	3	1	2	2	2	2
McCorkle et al. (2009)	3	2	1	3	3	3	3
Peters-Klimm et al. (2010)	2	3	1	3	3	2	2
Shearer et al. (2007)	3	1	1	1	2	1	3
Sorensen and Frich (2008)	3	3	1	2	3	3	3
Tsai et al. (2015)	2	2	1	2	3	3	2
Tsay et al. (2005)	3	3	1	3	3	3	3
Tummers et al. (2012)	2	2	1	1	3	3	3
Wagner et al. (2014)	2	3	1	3	2	2	3

^a^Bias. Author's judgement. 1 = High risk, 2 = Low risk, 3 = Unclear risk

After the codification, the intercoders agreement was 0.84 (Cohen’s Kappa mean was κ = 0.71, and the mean of the Spearman-Brown correlation was r = 0.97). Disagreements were solved through coders deliberation.

Ten overall ES estimations were performed, one for each of the subscales and summary measures included in the SF-36. Physical Functioning subscale was the one that was registered in the highest number of studies (n = 18).

**Fig 2:**
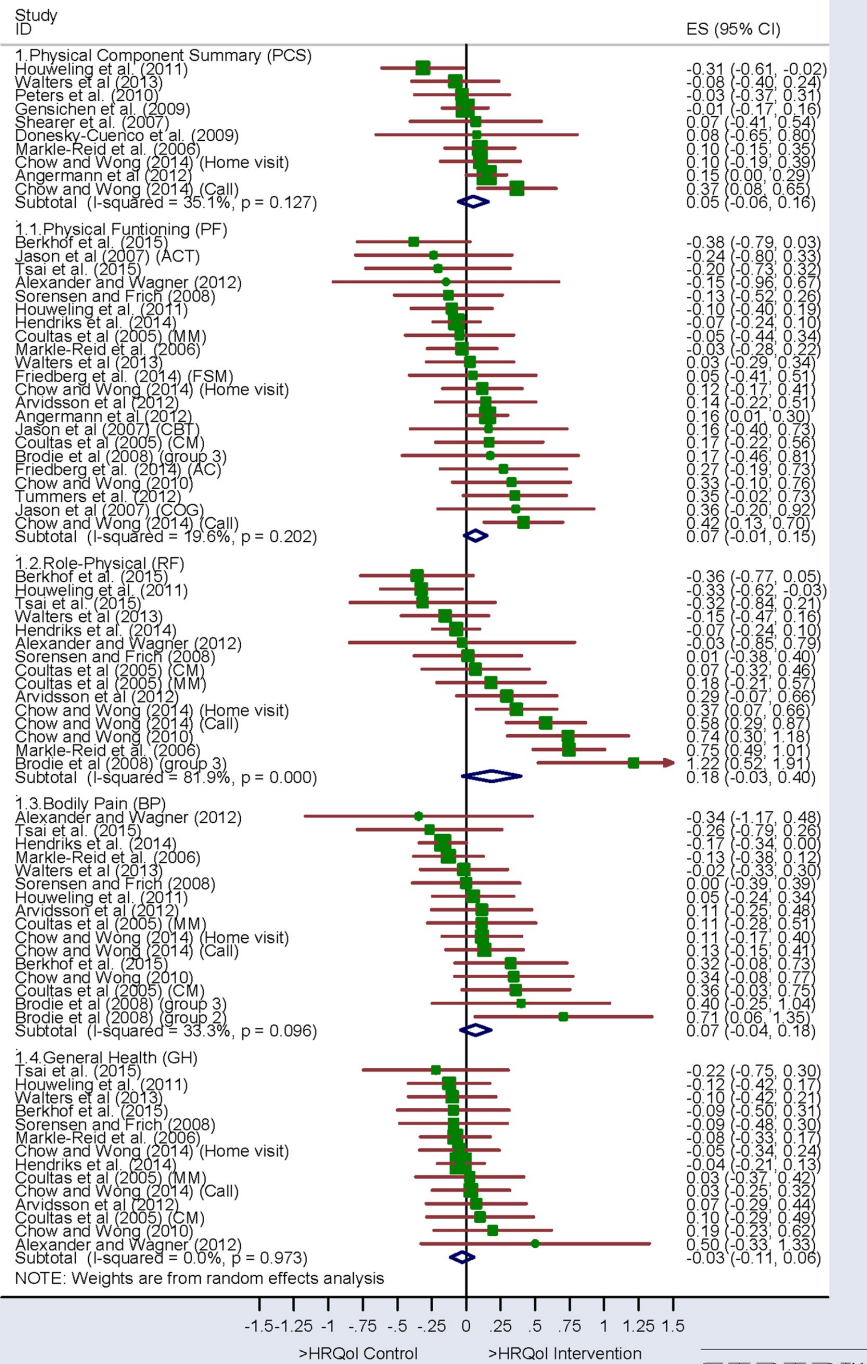
Forest plot. Physical dimensions (SF-36 and SF-12).

**Fig 3:**
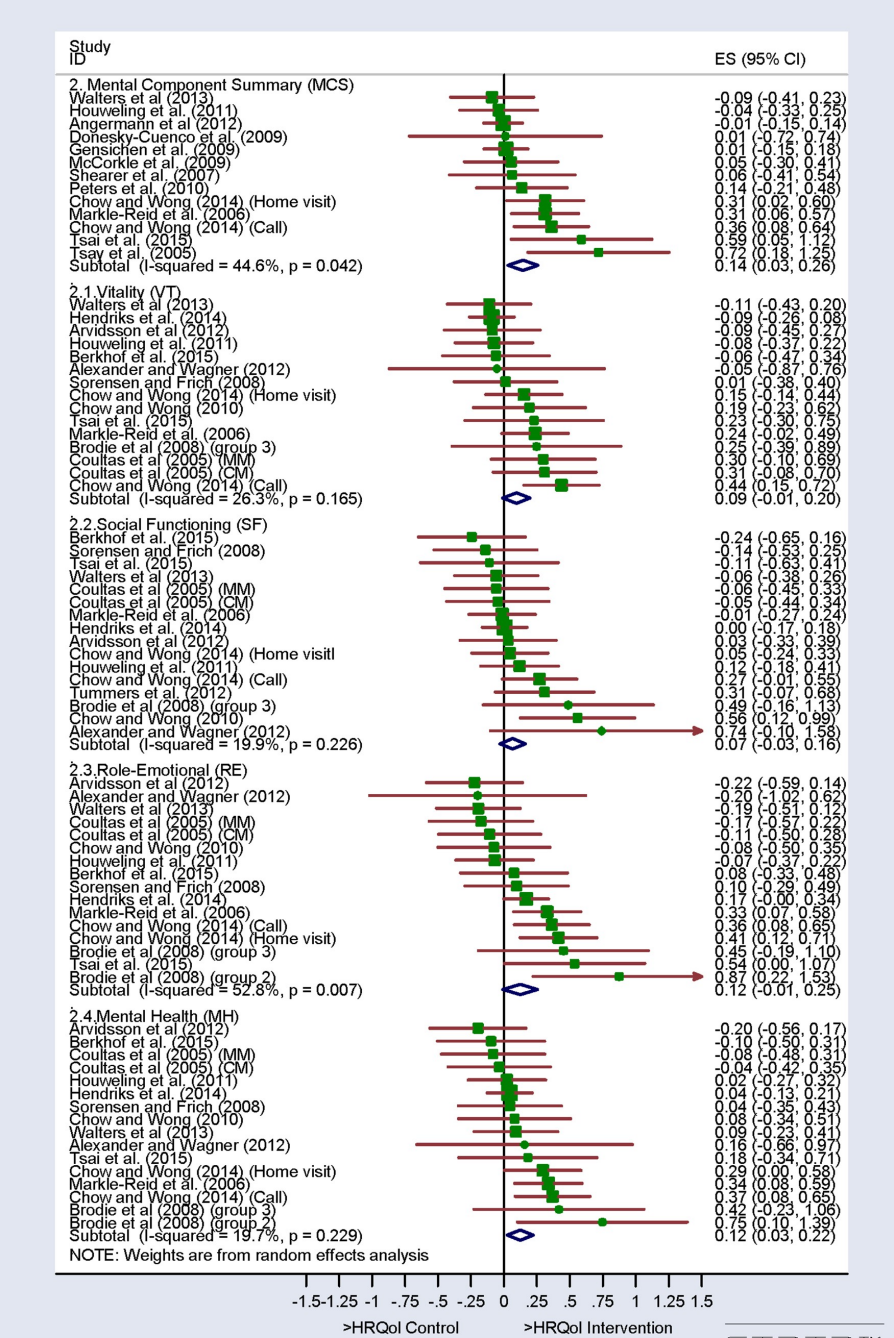
Forest plot. Mental dimensions (SF-36 and SF-12).

After eliminating the outliers detected in the sensitivity analysis, the overall ES varied between -0.06 and 0.40 (Figs 2 and 3), only obtaining a significant overall ES in the Mental Health subscale and the Mental Component Summary (Fig 3). The plus sign on the estimation indicates a score favorable to the intervention group, and a minus sign indicates a score favorable to the control group.

The scores from the Begg test (z = 1.26 p = 0.208), as well as those from the Egger test (bias = 1.34, p = 0.115) indicate lack of bias.

On scales where the overall ES was significant, this is, Mental Component Summary (MCS) measure and Mental Health subscale, bivariate analyses were conducted, which were used to explain the heterogeneity detected.

Among the variables analyzed, those that were found significant in the bivariate analyses are displayed in Table 4. Interventions on Treatment and Procedures (ES = 0.504; 95%CI: 0.129 − 0.879), and those on Case Management (ES = 0.241; 95%CI: 0.066 − 0.416) produced significant improvements on MCS measure. In addition, interventions of shorter duration (≤ 12 weeks) (ES = 0.346; 95%CI: 0.183 − 0.509), with follow-up period (ES = 0.384; 95%CI: 0.194 − 0.574) and based on a theory (ES = 0.331; 95%CI: 0.186 − 0.476) were significantly related to this measure. Multivariate models that were created with variables that were significant in the bivariate analyses are not shown because they lost their significance when included in these models.

**Table 4:** Bivariate analysis. Mixed effects. Mental Component Summary measure.

*Variables*		*k*	*ES*	*95%CI*		REML		
						*B Coefficient*	*p*	*R*^*2*^
Duration of the intervention								
	≤ 12 weeks	6	0.346	0.183	0.509			100.00%
	> 12 weeks	7	0.039	-0.048	0.126	-0.3075	0.0011	
Theory								
	Yes	5	0.331	0.186	0.476			99.98%
	No	8	0.020	-0.069	0.109	-0.3106	0.0003	
Follow-up								
	Yes	3	0.384	0.194	0.574			100.00%
	No	10	0.059	-0.033	0.151	-0.3315	0.0017	
Intervention classification								
	Teaching, Guidance an Counselling	3	-0.009	-0.220	0.203			100.00%
	Treatments and Procedures	3	0.504	0.129	0.879	0.5245	0.0096	
	Case Management	4	0.241	0.066	0.416	0.2528	0.0504	
	Surveillance	3	0.015	-0.090	0.120	0.0239	0.8424	

REML: Restrited Maximum Likelihood; *k*: Number of interventions for each outcome included in the anlaysis. ES: Overall Effect Size; CI: Confidence Interval; R^2^: Percentage of heterogeneity that the moderator accounts for.

## Discussion

This review summarized 24 clinical trials with interventions conducted by nurses aimed towards the increase of HRQoL of people with chronic diseases. Most studies produced an increase in HRQoL, although it was small and not always significant. Subgroup analyses supported the robustness of the overall conclusions. In this sense, subgroup analyses involving factors such as different sample sizes, participant characteristics, composition of the control group or correspondence of NCDs and intervention did not produce any qualitative difference in the small effect sizes.

Mental Health subscale and Mental Component Summary measure were the only scales where the overall effect size was significant. Although small, any improvement should be appreciated, as the majority of subjects studied are elderly patients with at least one chronic disease, from which a worsening of their HRQoL is expected. We believe the same is true for the Physical Component Summary. It is unlikely to improve the physical component of HRQoL as most studies target the elderly population with a chronic condition that increases disability with time. However, more tailored interventions with adequate follow-up, and designed for specific diseases may achieve better outcomes. The best results were obtained in studies lasting less than 12 weeks, that were based on a theory, that had a follow-up period, and when interventions were classified as “Treatment and procedures” or “Case management”.

This systematic review shows that nurses, especially in Western Europe and North America, have conducted interventions involving more than 4000 chronic patients aged 36 to 79 years. This highlights the involvement and interest of nursing in improving the quality of life of chronic patients. Data from clinical trials contribute to the methodological quality of the results obtained, favoring the generalization of their application in similar areas. Likewise, these results also bring to light the concern of the oldest countries with the need to analyze the phenomenon studied, as most projects were funded in order to implement actions that aim to reduce the impact of diseases, on the one hand [25,36,41–44] and to prevent the emergence of possible complications, on the other [28–30].

Among the chronic pathologies included in the studies selected, Chronic Obstructive Pulmonary Disease (COPD) was the most frequent, followed by heart failure, which was expected as these two are commonly among the most frequent NCDs [3,7,9,15] and are known to impair quality of life [49,50].

The duration of the interventions ranged widely among the different studies, oscillating from only four weeks to up to 104 weeks. Although this systematic review did not allow us to clearly conclude what duration was the best or most adequate, the analysis revealed that the most-effective interventions were those of the shortest duration, and especially those that had a follow-up period. This may have to do with the fact that compliance with both pharmacological and non-pharmacological therapies is generally low in chronic disease patients [51–53] and it even decreases over time [54,55], since, in short-term interventions, both patients and staff are more engaged in the process.

Interventions on treatments and procedures and on case management were not just the most common, but also those that had the greatest positive effect on HRQoL. In relation to the former, they have amply shown their effectiveness previously in cancer patients in a review of systematic reviews [56], but it is still to be confirmed their efficacy on other type of chronic patients. Case management has demonstrated to reduce the use of hospital services and improve the health of chronic patients [57], although a recent review shows that patients still perceive barriers such as lack of information and time constraints for accessing this type of health service [58]. Hudon et al. [59] recommended that case management interventions for frequent users of health services, such as chronic disease patients, ensure appropriate case finding processes and sufficient intervention intensity, among others. Weingarten et al. [60], in a meta-analysis on the management of disease in patients with chronic diseases, found that patient’s education was an integral part of most intervention programs. In fact, many of the interventions in this study were classified as "Case Management" or "Surveillance," but also included educational components. However, interventions that relied exclusively on "Teaching, Guidance, and Counseling" did not show improvement in HRQoL. Education may be a necessary but not sufficient condition for this purpose.

Although the interventions were applied to patients with long-term diseases, most of them were conducted without follow-up period which may have affected their efficacy. Also, it should be noted that most health professionals performed the intervention without it being based on any theory, and without specific training. The application of a theory for the intervention design and the evaluation of complex interventions of behavioral change is considered a good practice, but in spite of this, most studies omitted if they were based on a theory, as also pointed out in some reviews [61,62].

The results obtained in the meta-analysis indicate that interventions did not result in a significant improvement of the HRQoL evaluated with the SF-36, except in the Mental Health subscale and Mental Component Summary measure, where, in any case, the impact on HRQoL was very small, in agreement with similar systematic reviews [8,9,12,63,64].

As for the biases assessed according to the Cochrane guidelines [22], the control of the random sequence generation was generally good, while in many cases, there was not sufficient information for the evaluation of other items such as “blinding of participants and personnel” or “blinding of outcome assessment”, so bias was classified as unclear. Due to the nature of the interventions, patient or personnel blinding was not possible. However, the degree of bias is unknown. Future experimental studies on this matter should provide the minimum necessary information on the efforts made to avoid bias.

Several limitations to this study are noteworthy. The heterogeneity when grouping the data, as well as the possible existence of unexplored confounding explanatory factors led us to be cautious when establishing some variables mentioned as real predictive factors for the improvement of HRQoL. The exclusion of articles published in languages other than Spanish or English, and the scarcity of statistical data or information provided by some of the selected papers are other limitations that could also imply that some research results could have been ignored. Thus, the addition of new studies is advisable in order to reach more accurate conclusions.

## Conclusions

Worldwide, nurses perform multiple interventions destined to people with chronic diseases, in which HRQoL assessment is common. These interventions are too heterogeneous, and although they produced an overall improvement of the HRQoL favorable to the group interventions, this was small, and in many occasions not significant.

The results obtained in the meta-analysis indicated that except for Mental Health subscale and Mental Component Summary measure, the interventions did not achieve a significant improvement of HRQoL as evaluated by the SF-36. Also, although the overall size effect in Mental Health subscale was favorable and significant to the intervention group, it was small, according to Cohen’s classification.

It is proposed that further studies assessing HRQoL should conduct interventions, if possible on case management and treatments and procedures, based on a theoretical framework, adequately controlling bias with an exhaustive description of the data and its analysis, including an satisfactory follow-up and previous training of the health professionals.

One of the obstacles to the visibility of nursing is that, in many contexts, it is not recognized as a rigorous scientific discipline. For this reason, we believe that revisions such as this one help to make the contribution of nurses measurable and visible.

## Supporting information

S1 File Coding manual, data extraction sheet and work procedure. (PDF)

S2 File Characteristics of the interventions. (PDF)

S3 File Additional statistical analysis information. (PDF)

S4 File PRISMA checklist. (PDF)

## References

[1] WHO. Non-communicable diseases [Internet]. 2018 Available from: https://www.who.int/news-room/fact-sheets/detail/noncommunicable-diseases

[2] Chapel JM, Ritchey MD, Zhang D, Wang G. Prevalence and Medical Costs of Chronic Diseases Among Adult Medicaid Beneficiaries. Am J Prev Med. 2017 12;53(6):S143–54. 29153115 10.1016/j.amepre.2017.07.019 PMC5798200

[3] Vos T, Barber RM, Bell B, Bertozzi-Villa A, Biryukov S, Bolliger I, et al Global, regional, and national incidence, prevalence, and years lived with disability for 301 acute and chronic diseases and injuries in 188 countries, 1990–2013: a systematic analysis for the Global Burden of Disease Study 2013. Lancet. 2015 8;386(9995):743–800. 10.1016/S0140-6736(15)60692-4 26063472 PMC4561509

[4] Pellisé F, Vila-Casademunt A, Ferrer M, Domingo-Sàbat M, Bagó J, Pérez-Grueso FJS, et al Impact on health related quality of life of adult spinal deformity (ASD) compared with other chronic conditions. Eur Spine J. 2015 1;24(1):3–11. 10.1007/s00586-014-3542-1 25218732

[5] Ibrahim N, Teo SSL, Che Din N, Abdul Gafor AH, Ismail R. The Role of Personality and Social Support in Health-Related Quality of Life in Chronic Kidney Disease Patients. Remuzzi G, editor. PLoS One. 2015 7;10(7):e0129015 10.1371/journal.pone.0129015 26131714 PMC4488553

[6] Heins MJ, Korevaar JC, Hopman PEPC, Donker GA, Schellevis FG, Rijken MPM. Health-related quality of life and health care use in cancer survivors compared with patients with chronic diseases. Cancer. 2016 3;122(6):962–70. 10.1002/cncr.29853 26748907

[7] Bauer UE, Briss PA, Goodman RA, Bowman BA. Prevention of chronic disease in the 21st century: elimination of the leading preventable causes of premature death and disability in the USA. Lancet. 2014 7;384(9937):45–52. 10.1016/S0140-6736(14)60648-6 24996589

[8] Kadu MK, Stolee P. Facilitators and barriers of implementing the chronic care model in primary care: a systematic review. BMC Fam Pract. 2015 12;16(1):12. 25655401 10.1186/s12875-014-0219-0 PMC4340610

[9] Sav A, King MA, Whitty JA, Kendall E, McMillan SS, Kelly F, et al Burden of treatment for chronic illness: a concept analysis and review of the literature. Heal Expect. 2015 6;18(3):312–24. 10.1111/hex.12046 PMC5060781 23363080

[10] WHO. Global strategic directions for strengthening nursing and midwifery 2016–2020 [Internet]. 2016 Available from: https://www.who.int/hrh/nursing_midwifery/global-strategic-midwifery2016-2020.pdf?ua=1

[11] Shumaker SA, M. N. The international assessment of health-related quality of life: a theoretical perspective In: Shumaker S, Berton R, editors. The International assessment of health-related quality of life: theory, translation, measurement and analysis, Rapid Communications. Rapid Communications of Oxford; 1995 p. 3–10.

[12] Bakas T, McLennon SM, Carpenter JS, Buelow JM, Otte JL, Hanna KM, et al Systematic review of health-related quality of life models. Health Qual Life Outcomes. 2012 11;10(1):134. 23158687 10.1186/1477-7525-10-134 PMC3548743

[13] Devins GM. Using the Illness Intrusiveness Ratings Scale to understand health-related quality of life in chronic disease. J Psychosom Res. 2010 6;68(6):591–602. 10.1016/j.jpsychores.2009.05.006 20488277

[14] Blakemore A, Dickens C, Guthrie E, Bower P, Kontopantelis E, Afzal C, et al Depression and anxiety predict health-related quality of life in chronic obstructive pulmonary disease: systematic review and meta-analysis. Int J Chron Obstruct Pulmon Dis. 2014 5;9:501 10.2147/COPD.S58136 24876770 PMC4035108

[15] Jonkman NH, Schuurmans MJ, Groenwold RHH, Hoes AW, Trappenburg JCA. Identifying components of self-management interventions that improve health-related quality of life in chronically ill patients: Systematic review and meta-regression analysis. Patient Educ Couns. 2016 7;99(7):1087–98. 10.1016/j.pec.2016.01.022 26856778

[16] Brazier JE, Harper R, Jones NM, O’Cathain A, Thomas KJ, Usherwood T, et al Validating the SF-36 health survey questionnaire: new outcome measure for primary care. BMJ. 1992 7;305(6846):160–4. 10.1136/bmj.305.6846.160 1285753 PMC1883187

[17] Ware JE. SF-36 health survey update. Spine (Phila Pa 1976). 2000 12;25(24):3130–9. 11124729 10.1097/00007632-200012150-00008

[18] Revicki DA, Rentz AM, Luo MP, Wong RL. Psychometric characteristics of the short form 36 health survey and functional assessment of chronic illness Therapy-Fatigue subscale for patients with ankylosing spondylitis. Health Qual Life Outcomes. 2011;9:36 10.1186/1477-7525-9-36 21600054 PMC3124410

[19] Russo J, Trujillo CA, Wingerson D, Decker K, Ries R, Wetzler H, et al The MOS 36-Item Short Form Health Survey: reliability, validity, and preliminary findings in schizophrenic outpatients. Med Care. 1998;36(5):752–6. 9596066 10.1097/00005650-199805000-00015

[20] Bunevicius A. Reliability and validity of the SF-36 Health Survey Questionnaire in patients with brain tumors: a cross-sectional study. Health Qual Life Outcomes. 2017;15(1):91–2. 10.1186/s12955-017-0668-y 28472964 PMC5418840

[21] Treanor C, Donnelly M. A methodological review of the Short Form Health Survey 36 (SF-36) and its derivatives among breast cancer survivors. Qual Life Res. 2015;24(2):339–62. 10.1007/s11136-014-0785-6 25139502

[22] Higgins JPT, Green S. Cochrane handbook for systematic reviews of interventions Version 5.1. 0. The Cochrane Collaboration, 2011 Vol. 2011, www.cochrane-handbook.org. 2011.

[23] Intervention Scheme [Internet]. The Omaha System. 2018 Available from: http://www.omahasystem.org/interventionscheme.html

[24] Alexander JL, Wagner CL. Is Harmonica Playing an Effective Adjunct Therapy to Pulmonary Rehabilitation? Rehabil Nurs. 2012;37(4):207–12. 10.1002/rnj.33 22744994

[25] Angermann CE, Stoerk S, Gelbrich G, Faller H, Jahns R, Frantz S, et al Mode of Action and Effects of Standardized Collaborative Disease Management on Mortality and Morbidity in Patients With Systolic Heart Failure The Interdisciplinary Network for Heart Failure (INH) Study. Circ Fail. 2012;5(1):25–U109. 10.1161/CIRCHEARTFAILURE.111.962969 21956192

[26] Gellis ZD, Kenaley BL, Ten Have T. Integrated Telehealth Care for Chronic Illness and Depression in Geriatric Home Care Patients: The Integrated Telehealth Education and Activation of Mood (I-TEAM) Study. J Am Geriatr Soc. 2014;62(5):889–95. 10.1111/jgs.12776 24655228

[27] Gensichen J, von Korff M, Peitz M, Muth C, Beyer M, Guthlin C, et al Case management for depression by health care assistants in small primary care practices: a cluster randomized trial. Ann Intern Med. 2009;151(6):369–78. 19755362 10.7326/0003-4819-151-6-200909150-00001

[28] Hendriks JML, Vrijhoef HJM, Crijns HJGM, Brunner-La Rocca HP. The effect of a nurse-led integrated chronic care approach on quality of life in patients with atrial fibrillation. Europace. 2014;16(4):491–9. 24058179 10.1093/europace/eut286

[29] Houweling ST, Kleefstra N, van Hateren KJ, Groenier KH, Meyboom-de Jong B, Bilo HJ. Can diabetes management be safely transferred to practice nurses in a primary care setting? A randomised controlled trial. J Clin Nurs. 2011;20(9–10):1264–72. 10.1111/j.1365-2702.2010.03562.x 21401764

[30] Jason LA, Torres-Harding S, Friedberg F, Corradi K, Njoku MG, Donalek J, et al Non-pharmacologic interventions for CFS: A randomized trial. J Clin Psychol Med Settings. 2007;14(4):275–96.

[31] Markle-Reid M, Weir R, Browne G, Roberts J, Gafni A, Henderson S. Health promotion for frail older home care clients. J Adv Nurs. 2006;54(3):381–95. 10.1111/j.1365-2648.2006.03817.x 16629922

[32] McCorkle R, Dowd M, Ercolano E, Schulman-Green D, Williams AL, Siefert ML, et al Effects of a nursing intervention on quality of life outcomes in post-surgical women with gynecological cancers. Psychooncology. 2009;18(1):62–70. 10.1002/pon.1365 18570223 PMC4186244

[33] Peters-Klimm F, Campbell S, Hermann K, Kunz CU, Mueller-Tasch T, Szecsenyi J, et al Case management for patients with chronic systolic heart failure in primary care: The HICMan exploratory randomised controlled trial. Trials. 2010;11:56 10.1186/1745-6215-11-56 20478035 PMC2882359

[34] Shearer NBC, Cisar N, Greenberg EA. A telephone-delivered empowerment intervention with patients diagnosed with heart failure. Hear Lung. 2007;36(3):159–69. 10.1016/j.hrtlng.2006.08.006 17509423

[35] Sorensen J, Frich L. Home visits by specially trained nurses after discharge from multi-disciplinary pain care: A cost consequence analysis based on a randomised controlled trial. Eur J Pain. 2008;12(2):164–71. 10.1016/j.ejpain.2007.04.004 17560819

[36] Arvidsson S, Bergman S, Arvidsson B, Fridlund B, Tingstrom P. Effects of a self-care promoting problem-based learning programme in people with rheumatic diseases: a randomized controlled study. J Adv Nurs. 2013;69(7):1500–14. 10.1111/jan.12008 22973890

[37] Tsai S-H, Wang M-Y, Miao N-F, Chian P-C, Chen T-H, Tsai P-S. The Efficacy of a Nurse-Led Breathing Training Program in Reducing Depressive Symptoms in Patients on Hemodialysis: A Randomized Controlled Trial. Am J Nurs. 2015;115(4):24–42. 10.1097/01.NAJ.0000463023.48226.16 25793429

[38] Tsay SL, Lee YC, Lee YC. Effects of an adaptation training programme for patients with end-stage renal disease. J Adv Nurs. 2005;50(1):39–46. 10.1111/j.1365-2648.2004.03347.x 15788064

[39] Tummers M, Knoop H, van Dam A, Bleijenberg G. Implementing a minimal intervention for chronic fatigue syndrome in a mental health centre: a randomized controlled trial. Psychol Med. 2012;42(10):2205–15. 10.1017/S0033291712000232 22354999

[40] Walters J, Cameron-Tucker H, Wills K, Schuez N, Scott J, Robinson A, et al Effects of telephone health mentoring in community-recruited chronic obstructive pulmonary disease on self-management capacity, quality of life and psychological morbidity: a randomised controlled trial. BMJ Open. 2013;3(9):e003097–e003097. 10.1136/bmjopen-2013-003097 24014482 PMC3773640

[41] Berkhof FF, van den Berg JWK, Uil SM, Kerstjens HAM. Telemedicine, the effect of nurse-initiated telephone follow up, on health status and health-care utilization in COPD patients: A randomized trial. Respirology. 2015;20(2):279–85. 10.1111/resp.12437 25400242

[42] Brodie DA, Inoue A, Shaw DG. Motivational interviewing to change quality of life for people with chronic heart failure: a randomised controlled trial. Int J Nurs Stud. 2008;45(4):489–500. 10.1016/j.ijnurstu.2006.11.009 17258218

[43] Chow SKY, Wong FK. Health-related quality of life in patients undergoing peritoneal dialysis: effects of a nurse-led case management programme. J Adv Nurs. 2010;66(8):1780–92. 10.1111/j.1365-2648.2010.05324.x 20557392

[44] Chow SKY, Wong FKY. A randomized controlled trial of a nurse-led case management programme for hospital-discharged older adults with co-morbidities. J Adv Nurs. 2014;70(10):2257–71. 10.1111/jan.12375 24617755 PMC4263097

[45] Coultas D, Frederick J, Barnett B, Singh G, Wludyka P. A randomized trial of two types of nurse-assisted home care for patients with COPD. Chest. 2005;128(4):2017–24. 10.1378/chest.128.4.2017 16236850

[46] Donesky-Cuenco D, Nguyen HQ, Paul S, Carrieri-Kohlman V. Yoga therapy decreases dyspnea-related distress and improves functional performance in people with chronic obstructive pulmonary disease: a pilot study. J Altern Complement Med. 2009;15(3):225–34. 10.1089/acm.2008.0389 19249998 PMC3051406

[47] Friedberg F, Napoli A, Coronel J, Adamowicz J, Seva V, Caikauskaite I, et al Chronic Fatigue Self-Management in Primary Care: A Randomized Trial. Psychosom Med. 2013;75(7):650–7. 10.1097/PSY.0b013e31829dbed4 23922399 PMC3785003

[48] Moher D, Liberati A, Tetzlaff J, Altman DG, Group P. Preferred reporting items for systematic reviews and meta-analyses: the PRISMA statement. PLoS Med. 2009;6(7):e1000097 10.1371/journal.pmed.1000097 19621072 PMC2707599

[49] Hobbs FDR, Kenkre JE, Roalfe AK, Davis RC, Hare R, Davies MK. Impact of heart failure and left ventricular systolic dysfunction on quality of life: a cross-sectional study comparing common chronic cardiac and medical disorders and a representative adult population. Eur Heart J. 2002 12;23(23):1867–76. 10.1053/euhj.2002.3255 12445536

[50] Tselebis A, Pachi A, Ilias I, Kosmas E, Bratis D, Moussas G, et al Strategies to improve anxiety and depression in patients with COPD: a mental health perspective. Neuropsychiatr Dis Treat. 2016;12:297–328. 10.2147/NDT.S79354 26929625 PMC4755471

[51] van der Wal MHL, van Veldhuisen DJ, Veeger NJGM, Rutten FH, Jaarsma T. Compliance with non-pharmacological recommendations and outcome in heart failure patients. Eur Heart J. 2010 6;31(12):1486–93. 10.1093/eurheartj/ehq091 20436049

[52] Park K, Cho S, Bower JK. Changes in Adherence to Non-Pharmacological Guidelines for Hypertension. PLoS One. 2016;11(8):e0161712 10.1371/journal.pone.0161712 27561006 PMC4999088

[53] Seid MA, Abdela OA, Zeleke EG. Adherence to self-care recommendations and associated factors among adult heart failure patients. From the patients’ point of view. PLoS One. 2019;14(2):e0211768 10.1371/journal.pone.0211768 30730931 PMC6366768

[54] Maeder A, Poultney N, Morgan G, Lippiatt R. Patient Compliance in Home-Based Self-Care Telehealth Projects. J Telemed Telecare. 2015 12;21(8):439–42. 10.1177/1357633X15612382 26556057

[55] Ehrmann D, Spengler M, Jahn M, Niebuhr D, Haak T, Kulzer B, et al Adherence Over Time: The Course of Adherence to Customized Diabetic Insoles as Objectively Assessed by a Temperature Sensor. J Diabetes Sci Technol. 2018 5;12(3):695–700. 10.1177/1932296817747618 29281893 PMC6154238

[56] Duncan M, Moschopoulou E, Herrington E, Deane J, Roylance R, Jones L, et al Review of systematic reviews of non-pharmacological interventions to improve quality of life in cancer survivors. BMJ Open. 2017 11;7(11):e015860 10.1136/bmjopen-2017-015860 29187408 PMC5719270

[57] Joo JY, Liu MF. Case management effectiveness in reducing hospital use: a systematic review. Int Nurs Rev. 2017 6;64(2):296–308. 10.1111/inr.12335 27861853

[58] Joo JY, Liu MF. Experiences of case management with chronic illnesses: a qualitative systematic review. Int Nurs Rev. 2018 3;65(1):102–13. 10.1111/inr.12429 29336031

[59] Hudon C, Chouinard M-C, Lambert M, Diadiou F, Bouliane D, Beaudin J. Key factors of case management interventions for frequent users of healthcare services: a thematic analysis review. BMJ Open. 2017 10;7(10):e017762 10.1136/bmjopen-2017-017762 29061623 PMC5665285

[60] Weingarten SR, Henning JM, Badamgarav E, Knight K, Hasselblad V, Gano Jr A, et al Interventions used in disease management programmes for patients with chronic illness-which ones work? Meta-analysis of published reports. BMJ. 2002;325(7370):925 10.1136/bmj.325.7370.925 12399340 PMC130055

[61] Prestwich A, Sniehotta FF, Whittington C, Dombrowski SU, Rogers L, Michie S. Does theory influence the effectiveness of health behavior interventions? Meta-analysis. Health Psychol. 2014;33(5):465–74. 10.1037/a0032853 23730717

[62] Davies P, Walker AE, Grimshaw JM. A systematic review of the use of theory in the design of guideline dissemination and implementation strategies and interpretation of the results of rigorous evaluations. Implement Sci. 2010;5:14 10.1186/1748-5908-5-14 20181130 PMC2832624

[63] Bauer M, Fetherstonhaugh D, Haesler E, Beattie E, Hill KD, Poulos CJ. The impact of nurse and care staff education on the functional ability and quality of life of people living with dementia in aged care: A systematic review. Nurse Educ Today. 2018 8;67:27–45. 10.1016/j.nedt.2018.04.019 29729501

[64] Kivela K, Elo S, Kyngas H, Kaariainen M. The effects of health coaching on adult patients with chronic diseases: a systematic review. Patient Educ Couns. 2014 11;97(2):147–57. 10.1016/j.pec.2014.07.026 25127667

