# Supplementary material for: Characteristics of nursing interventions that improve the quality of life of people with chronic diseases. A systematic review with meta-analysis
Source: PLoS One. 2019 Jun 24;14(6):e0218903. doi: 10.1371/journal.pone.0218903 (PMC6590814; doi:10.1371/journal.pone.0218903)
Supplement: S1 File — (PDF) [file pone.0218903.s001.pdf]

PROTOCOL SYSTEMATIC REVIEW

INTERVENTIONS AIMED AT IMPROVING THE HEALTH RELATED QUALITY OF LIFE OF PEOPLE WITH CHRONIC DISEASES

The objective of this protocol is to extract, from selected primary studies, the information needed to conduct a systematic review, and if possible a meta-analysis, of interventions conducted by nurses aimed at improving the quality of life of people with chronic diseases.

Seven dimensions were created to group the information collected from the selected studies:

A. Identification
B. Design
C. Participants
D. Intervention
E. Baseline data
F. Main results
G. Biases
H. Observations

CHRONOGRAM

A minimum time of 15 months is estimated to carry out this review in which the following tasks are included:

1. Between March-3-2017 to May-1-2017:
    a. Establish the group of researchers
    b. Pose the research question
    c. Establish the exclusion inclusion criteria
    d. Define the search strategy
    e. Outline the protocol that will follow the systematic review

2. Between May-2-2017 to August-30-2017:
    a. Conduct the search and selection of studies
    b. Conclude the drafting of the protocol
    c. To elaborate, following the protocol, a data collection sheet in google.docs

3. Between August-30-2017 to September-15-2017:
    a. Carry out a pilot coding, with 2 -3 selected studies, intended to test the coding manual and the data collection sheet included in the protocol.
    b. Incorporate the suggested settings, after the pilot coding, into the protocol and the data collection sheet created in google.docs.

4. Between September-15-2017 and December-15-2017
    a. Coding, 2 researchers independently, i
    b. Create a database with coded information

5. Between December-15-2017 to April-1-2018
    a. Perform statistical analysis
    b. Describe the main results obtained
    c. Create tables and figures with the data

6. Between April-1-2018 to May-30-2018
    a. Writing a report

INTRODUCTION

The objective of the systematic review is to determine the efficacy of nursing interventions undertaken to improve the quality of life of people with chronic diseases.

The inclusion criteria of primary studies in the systematic review are as follows:

a. The study should aim at a nursing intervention aimed at improving the health-related quality of life in people >18 years diagnosed with chronic diseases.

b. The study design must be a clinical trial of at least two groups (experimental and control), in which the assignment to the groups is random..:
   1. Randomised clinical trial (RCT)/Randomised controled trial (RCT)
   2. RCT Cluster

c. The study should include at least two groups, one of which acts as a control group with pre-post measures in both groups.

d. The study must provide data from both the control group and the experimental group to determine the central tendency and pre and post dispersion scores of both groups, on all or some of the scales that make up the SF.

e. The study should be written in Spanish or English.

To carry out the coding of each of the selected studies this protocol includes two instruments:

1. A coding manual in which the guidelines to follow in the coding of each item are detailed.

2. A Data Collection Sheet created at docs.google.com, available at the following link https://goo.gl/forms/U33KB1Jg0ds6JDcD2, which can be filled in online. Although the sheet can also be printed and completed on paper, it is recommended that it be completed online, as this avoids the subsequent transcription of the data and reduces the errors derived from the transcription process. In the data collection sheet created in docs.google.com, accents, spaces and the letter "ñ" have been omitted so that when importing databases into statistical software such as R there is no problem with the recognition of certain characters (e.g. spaces, accents, ñ...).

CODING GUIDELINES

1º. Assign a code to each encoder.
2º. Read the coding protocol.
3º. Assign each primary study a number for easy identification within the study sample. Start with number 1 and number consecutively.
4º. Carefully read the study and simultaneously complete the data collection sheet following the explanations provided in this coding protocol.
5º. Write down, on the coding sheet, in red all the information extracted from the primary study that is doubtful.

A_IDENTIFICATION

G1_ ENCODER

Select the encoder.

G2_ STUDIO

Write down the number of the study.

G3_ AUTHOR

If several authors have participated in the study, note the surname of the main author followed by et al. and in brackets the year of publication on paper, e.g. Fernández et al. (2014).

If only 2 authors have participated in the study, note the surname of the main author followed by and the surname of the second author and in brackets the year of publication on paper, e.g. Fernández and Gómez. (2014).

G4_ DEPENDENCY

If an author has participated in another study, review the year of data collection from that other study and try to determine whether there is a sample dependency. Check "*Yes"* if a possible sample dependency is suspected, otherwise select "*No"*.

If sample dependence is detected among several studies, choose one and discard the rest. To select the study to choose, use the following criteria:

- The largest sample size
- The most current study
- The one that best suits the objectives of the review

G5_ TITLE

Write down the title of the study.

G6_ YEAR

Indicate the year of publication on paper, if the study is published. If the study is not published indicate the year in which it was presented to a Congress, read the thesis, etc.

G7_ DATA_COLLECTION_YEAR

Write down the start date of the data collection.

G8_ FUNDING

If the study has received funding indicate "*Yes*", if the study does not indicate anything concerning funding mark "*No*".

G9_ CONTINENT

Indicate the continent where the study was conducted: "*Africa*", "*North America*", "*South America*", "*Central America*", "*Asia*", "*Eastern Europe*", "*Western Europe*" or "*Oceania*".

G10_ COUNTRY

Note the country in which the study was conducted.

G11_ LANGUAGE

Mark the option that matches the language in which the primary study was written: "*Spanish*", "*English".*

G12_ SOURCE:

Mark with an X the corresponding option: "*Journal*", "*Book Chapter*", "*Thesis*", "*Work presented to Congress*", "*Unpublished Report*", "*Other*".

B_ DESIGN

G1_ TYPE

Select the appropriate option

"RCT" is usually translated as *randomized controlled trial* and *randomized clinical trial*. This type of design consists of treatments being randomly assigned to research subjects.

"Cluster RCT" the group of subjects (as opposed to the individual subjects) is randomly assigned to the treatment.

G2_ CENTER

Indicate the institution where the study was carried out: "*Consultation*", "*Hospital*", "*Multicentric*", "*Others*".

The terms "*Consultation*", "*Hospital*", imply a single Center, when the study involves more than one consultation or hospital is considered "*Multicentric*".

G3_ SHEET

Indicate the number of data collection sheets that have been recorded

It is customary to fill out one data collection sheet per study. However, in some studies more than two groups have participated, which means that more than one data collection sheet has to be filled in. For example if in a study there are 3 groups: Intervention A, Intervention B and Control 2 data collection sheets have to be filled in. One with data from group Intervention A and control and another data collection sheet with data from group Intervention B and control.

G4_ METHODS

Select the corresponding option "*Clinical Contact*", "*Announcement*", "*Other*".

C_PARTICIPANTS

G1_ EXPERIMENTAL

For persons participating in the experimental group, indicate the category in which they are included: "*Chronic*", "*Pluripathological*" or "*Main caregivers*".

G2_ CHRONIC_DISEASE

If in the previous question the option "Chronic" has been selected to write the pathology. If "*Pluripathological*" has been selected but all of them share the same chronic disease, e.g. HTA. indicate

G3_ CONTROL

For persons participating in the experimental group, indicate the category in which "*Chronic*", "*Pluripathological*", "*Main caregivers*", "*General population*" or "*Other*" are included.

D_INTERVENTION

G1_ DESCRIPTION

Write a brief description of the intervention

G2_ OBJECTIVE

Select the appropriate option

G3_ THEORY

If a theory has been followed mark "*Yes*", if nothing is indicated mark "*No*".

G4_ YES_THEORY

If in the previous question the option "Yes" has been checked, write here the name of the theory used.

G5_ SCALES

Write down the full name and in brackets the acronyms of all scales, questionnaires, tests... employed.

G6_ SCALE_QUALITY

Write down the full name and in brackets the abbreviations of the quality of life scale used.

G7_ SF_36/16

If the study has used the SF indicate whether it provides data from the 8 scales and the 2 two domains and the time in which the scores were collected (for example, pre and post measures).

G8_ WEEKS

Indicate, in weeks, the duration of the intervention.

1 year is 52 weeks and 6 months are 26 weeks.

G9_ SESSIONS

Indicate the number of sessions received by the intervention group.

G10_ MINUTES_SESION

Indicate the minutes that a session lasts in the intervention group.

G11_ MODALITY

Choose the appropriate option

G12_ ADDITIONAL_MATERIAL

If reinforcement material was used in the intervention such as written material, DVD... mark "*Yes*", if nothing is indicated mark "*No*".

G13_ CONTACT

Regarding the interaction between the professional and the person who participated in the intervention, indicate whether it was direct, indirect (e.g., via telephone, email, etc.) or a combination of both types of interaction.

G14_ INCENTIVE

If by participating in the study people were provided with some kind of incentive (e.g. money, bus trips to attend sessions, etc.) check "*Yes*"; otherwise "*No*".

G15_ TYPE

Select the type of interventions used. Options are not exclusive (more than one can be selected).

G16_ PROFESSIONAL

Choose the appropriate option on the profession of the person(s) who carried out the intervention (more than one can be selected).

G17_ SPECIFIC TRAINING

If in order to carry out the intervention the professional has received a previous specific training mark the option "*Yes*", otherwise indicate "*No*". If the study does not provide this information, mark "*No*".

G18_ CONTROL

Choose the appropriate option

G19_ FOLLOW-UP

If the study carried out follow-up, indicate "Yes", otherwise indicate "No". If the study does not provide this information, mark "No".

E_ BASAL DATA

Record the baseline data of the people who participated in the experimental group, as well as in the control group.

Except for age, which must provide the mean, for the rest of the variables note the frequency (the number of individuals).

If the study does not provide frequencies (*n*) but the %, transform that % to frequencies or also, in some cases it can be obtained by deduction. For example, if the study provides the number of women but not the number of men, subtracting the number of women from the sample size, the number of men who have participated in the study can be obtained.

Round decimal scores to two (if the decimal in third place is ≥5 add +1 to the decimal in second place and if, on the contrary, the decimal in third place is <5 leave the decimal in second place the same, e.g. 2,346 --> 2,35 ; 0,275 --> 0,28 ; 6,634-->6,63).

F_ RESULTS

Write down the main results described in the study as well as the time at which these results were obtained.

G_ BIASES

The guidelines in Chapter 8 of the Cochrane Handbook 5.1.0 will be followed to assess bias. (pg. 197-255)
https://es.cochrane.org/sites/es.cochrane.org/files/public/uploads/Manual_Cochrane_510_reduit.pdf
According to Cochrane, the bias is evaluated through 7 items each with three possible answers: "*Low*", "*High*" and "*Unclear*" risk.

G1_ RANDOM SEQUENCE GENERATION

Selection bias (biased allocation to interventions) due to inadequate generation of a randomised sequence.

Criteria for a judgment of 'Low risk' of bias. The investigators describe a random component in the sequence generation process such as:

- •Referring to a random number table;
- •Using a computer random number generator;
- •Coin tossing;
- •Shuffling cards or envelopes;
- •Throwing dice;
- •Drawing of lots;
- •Minimization*.
  *Minimization may be implemented without a random element, and this is considered to be equivalent to being random.

Criteria for the judgment of 'High risk' of bias. The investigators describe a non-random component in the sequence generation process. Usually, the description would involve some systematic, non-random approach, for example:

- Sequence generated by even or odd date of birth;
- Sequence generated by some rule based on the date (or day) of admission;
- Sequence generated by some rule based on hospital or clinic record number.

Other non-random approaches happen much less frequently than the systematic approaches mentioned above and tend to be obvious. They usually involve judgement or some method of non-random categorization of participants, for example:

- Allocation by judgement of the clinician;
- Allocation by preference of the participant;
- Allocation based on the results of a laboratory test or a series of tests;
- Allocation by availability of the intervention.

Criteria for the judgment of 'Unclear risk' of bias.

- Insufficient information about the sequence generation process to permit judgement of 'Low risk' or 'High risk'.

G2_ ALLOCATION CONCEALMENT

Selection bias (biased allocation to interventions) due to inadequate concealment of allocations prior to assignment.

Criteria for a judgement of '*Low*' risk of bias. Participants and investigators enrolling participants could not foresee assignment because one of the following, or an equivalent method, was used to conceal allocation:

- Central allocation (including telephone, web-based and pharmacy-controlled randomization);
- Sequentially numbered drug containers of identical appearance;
- Sequentially numbered, opaque, sealed envelopes.

Criteria for the judgment of 'High risk' of bias. Participants or investigators enrolling participants could possibly foresee assignments and thus introduce selection bias, such as allocation based on:

- Using an open random allocation schedule (e.g. a list of random numbers);
- Assignment envelopes were used without appropriate safeguards (e.g. if envelopes were unsealed or non-opaque or not sequentially numbered);
- Alternation or rotation;
- Date of birth;
- Case record number;
- Any other explicitly unconcealed procedure.

Criteria for the judgment of 'Unclear risk' of bias:

- Insufficient information to permit judgement of 'Low risk' or 'High risk'. This is usually the case if the method of concealment is not described or not described in sufficient detail to allow a definite judgement – for example if the use of assignment envelopes is described, but it remains unclear whether envelopes were sequentially numbered, opaque and sealed.

G3_ BLINDING OF PARTICIPANTS AND PERSONNEL

Performance bias due to knowledge of the allocated interventions by participants and personnel during the study.

Criteria for a judgment of 'Low risk' of bias. Any one of the following:

- No blinding or incomplete blinding, but the review authors judge that the outcome is not likely to be influenced by lack of blinding;
- Blinding of participants and key study personnel ensured, and unlikely that the blinding could have been broken.

Criteria for the judgment of 'High risk' of bias. Any one of the following:

- No blinding or incomplete blinding, and the outcome is likely to be influenced by lack of blinding;
- Blinding of key study participants and personnel attempted, but likely that the blinding could have been broken, and the outcome is likely to be influenced by lack of blinding.

Criteria for the judgment of 'Unclear risk' of bias. Any one of the following:

- Insufficient information to permit judgment of 'Low risk' or 'High risk';
- The study did not address this outcome.

G4_ BLINDING OF OUTCOME ASSESSMENT

Detection bias due to knowledge of the allocated interventions by outcome assessors.

Criteria for a judgment of ‘Low risk’ of bias. Any one of the following:

- No blinding of outcome assessment, but the review authors judge that the outcome measurement is not likely to be influenced by lack of blinding;
- Blinding of outcome assessment ensured, and unlikely that the blinding could have been broken.

Criteria for the judgment of ‘High risk’ of bias. Any one of the following:

- No blinding of outcome assessment, and the outcome measurement is likely to be influenced by lack of blinding;
- Blinding of outcome assessment, but likely that the blinding could have been broken, and the outcome measurement is likely to be influenced by lack of blinding.

Criteria for the judgment of ‘Unclear risk’ of bias. Any one of the following:

- Insufficient information to permit judgment of ‘Low risk’ or ‘High risk’;
- The study did not address this outcome.

G5_ INCOMPLETE OUTCOME DATA

Attrition bias due to amount, nature or handling of incomplete outcome data.

Criteria for a judgment of ‘Low risk’ of bias. Any one of the following:

- No missing outcome data;
- Reasons for missing outcome data unlikely to be related to true outcome (for survival data, censoring unlikely to be introducing bias);
- Missing outcome data balanced in numbers across intervention groups, with similar reasons for missing data across groups;
- For dichotomous outcome data, the proportion of missing outcomes compared with observed event risk not enough to have a clinically relevant impact on the intervention effect estimate;
- For continuous outcome data, plausible effect size (difference in means or standardized difference in means) among missing outcomes not enough to have a clinically relevant impact on observed effect size;
- Missing data have been imputed using appropriate methods.

Criteria for the judgment of ‘High risk’ of bias. Any one of the following:

- Reason for missing outcome data likely to be related to true outcome, with either imbalance in numbers or reasons for missing data across intervention groups;
- For dichotomous outcome data, the proportion of missing outcomes compared with observed event risk enough to induce clinically relevant bias in intervention effect estimate;
- For continuous outcome data, plausible effect size (difference in means or standardized difference in means) among missing outcomes enough to induce clinically relevant bias in observed effect size;
- ‘As-treated’ analysis done with substantial departure of the intervention received from that assigned at randomization;
- Potentially inappropriate application of simple imputation.

Criteria for the judgement of '*Unclear*' risk of bias. Any one of the following:

- Insufficient reporting of attrition/exclusions to permit judgement of ‘Low risk’ or ‘High risk’ (e.g. number randomized not stated, no reasons for missing data provided);
- The study did not address this outcome.

G6_ SELECTIVE REPORTING

Reporting bias due to selective outcome reporting.

Criteria for a judgment of 'Low risk' of bias. Any one of the following:

- The study protocol is available and all of the study's pre-specified (primary and secondary) outcomes that are of interest in the review have been reported in the pre-specified way;
- The study protocol is not available but it is clear that the published reports include all expected outcomes, including those that were pre-specified (convincing text of this nature may be uncommon).

Criteria for the judgment of 'High risk' of bias. Any one of the following:

- Not all of the study's pre-specified primary outcomes have been reported;
- One or more primary outcomes is reported using measurements, analysis methods or subsets of the data (e.g. subscales) that were not pre-specified;
- One or more reported primary outcomes were not pre-specified (unless clear justification for their reporting is provided, such as an unexpected adverse effect);
- One or more outcomes of interest in the review are reported incompletely so that they cannot be entered in a meta-analysis;
- The study report fails to include results for a key outcome that would be expected to have been reported for such a study.

Criteria for the judgment of 'Unclear risk' of bias:

- Insufficient information to permit judgement of 'Low risk' or 'High risk'. It is likely that the majority of studies will fall into this category.

G7_ Other bias

Bias due to problems not covered elsewhere in the table.

Criteria for a judgment of 'Low risk' of bias.

- The study appears to be free of other sources of bias.

Criteria for the judgment of 'High risk' of bias. There is at least one important risk of bias. For example, the study:

- Had a potential source of bias related to the specific study design used; or
- Has been claimed to have been fraudulent; or
- Had some other problem.

Criteria for the judgment of 'Unclear risk' of bias. There may be a risk of bias, but there is either:

- Insufficient information to assess whether an important risk of bias exists; or
  Insufficient rationale or evidence that an identified problem will introduce bias.

H_ Observations

Note any aspects that have not been included in the coding table (or that may be confusing) and that should be highlighted.
