# Supplementary material for: Characteristics of nursing interventions that improve the quality of life of people with chronic diseases. A systematic review with meta-analysis
Source: PLoS One. 2019 Jun 24;14(6):e0218903. doi: 10.1371/journal.pone.0218903 (PMC6590814; doi:10.1371/journal.pone.0218903)
Supplement: S2 File — (PDF) [file pone.0218903.s002.pdf]

Study	*Description of the intervention*	*Type of the intervention*
1. Alexander and Wagner (2012), USA	Sessions of physical conditioning were combined with at-home exercises with the harmonica, as a tool for fomenting the practice of breathing exercises of inhalation and exhalation.	2
2. Angermann et al. (2012), Germany	The program includes monitoring and education of the patient in a collaborative focus that involves nurses, general practitioners and cardiologists, and training and supervision of the caregivers. The first contact is conducted at the hospital with phone follow-ups.	4
3. Arvidsson et al. (2012), Sweden	The intervention consisted in self-directed learning divided into 7 tutorials, where a subject was presented, followed by a discussion among the participants where they discussed their experience, contributed their thoughts, searched for information, applied the new concepts to their life and evaluated them.	1
4. Berkhof et al. (2015), Netherlands	Interventions through phone calls, which consisted on structured calls that included a brief introduction and where two questionnaires were administered, destined to detect symptoms.	3
5. Brodie et al. (2008), United Kingdom	Two types of interventions were compared, besides the control group (group1). In group 2, a motivational interview was employed to integrate physical activities into the patient's routine. In group 3, the motivational interview is combined with regular care.	2
6. Chow and Wong (2010), China	Comprehensive education program before hospital discharge and standardized phone call follow-up.	4
7. Chow and Wong (2014), China	Two types of interventions were compared (Home visit and call), besides the control group. In the Home visits, the visit to the patient's home was combined with the telephone call. In the Call intervention, only calls were made with the same objectives as the Home visit.	4
8. Coultas et al. (2005), USA	There are two intervention groups: nurse-assisted medical management (MM) and nurse-assisted collaborative management (CM); in both, 8 hours are provided for nurses, but although in the MM this was focused on the management of COPD, in CM, it was focused on the development of collaborative care.	3
9. Donesky-Cuenco et al. (2009), USA	A yoga program designed specifically for persons with COPD, given two times per week that included asanas (yoga poses) and visama vritti pranayama (timed respiration).	2
10. Friedberg et al. (2013), USA	It compares two intervention groups and one control group. In the interventions, it combines educational intervention with self-management and change of cognitive behavior or the monitoring of symptoms.	4
11. Gellis et al. (2014), USA	Daily tele-monitoring of symptoms, body weight and use of medicines, and weekly sessions for the resolution of depression problems. Also, it provided information to the primary attention doctors.	4
12. Gensichen et al. (2009), Germany	Phone interview structured towards the control of symptoms of depression and support for the compliance of medication, with comments for the family doctor.	4
13. Hendriks et al. (2014), Netherlands	Intervention directed by nurses, which consisted on care based on guides and supported by software, and supervised by cardiologists.	3
14. Houweling et al. (2011), Netherlands	Intervention destined to provide practical education to nurses so that they would be able to provide diabetic care without supervision.	3
15. Jason et al. (2007), USA	Four groups were compared, who received different types of non-pharmacologic therapy: cognitive behavior, cognitive treatment, anaerobic activity or relaxation.	2

16. Markle-Reid et al. (2006), Canada	Evaluation of health combined with regular house visits or telephone contact, health education on the management of disease, coordination of community services and the use of empowerment strategies to improve independency.	3
17. McCorkle et al. (2009), USA	Intervention destined to the development and maintenance of skills for the post-operational self-management and the enabling of active participation in the decisions that affect later treatment (chemotherapy).	1
18. Peters-Klimm et al. (2010), Germany	"Case management", consisted on the management of cases, which combined education on the disease, phone follow-up destined to detect signs of alarm and adherence, as well as home visits.	4
19. Shearer et al. (2007), USA	At discharge, telephone calls are conducted following a pre-established script, where the patient is provided with support and information that provide him or her with empowerment, awareness and self-management of his or her disease.	1
20. Sorensen and Frich (2008), Denmark	Home visits by the nurse once the patient is discharged, and every 4 months for 2 years, where, besides educating about pharmacotherapy, pain and sleeping problems, secondary effects and coping strategies, adherence is re-enforced.	1
21. Tsai et al. (2015), Taiwan	Training with respiratory exercises.	2
22. Tsay et al. (2005), Taiwan	The program included education, the evaluation of needs, and the modification of cognitive behavior, problem resolution and management of stress. For the training portion, role-playing was utilized.	2
23. Tummers et al. (2012), Netherlands	Cognitive-behavioral therapy as guided self-instruction through the use of a manual. The patients provided information about their changes and doubts through e-mail.	2
24. Walters et al. (2013), Australia	Intervention that combined cognitive-type training directed to the nurse and regular phone calls to the patients which aimed to ease the management of health problems and health behaviors.	1

Each intervention was categorized in one of the four categories of the Omaha System intervention classification:

1. Teaching, Guidance, and Counseling: Activities designed to provide information and materials, encourage action and responsibility for self-care and coping, and assist the individual/family/community to make decisions and solve problems.
2. Treatments and Procedures: Technical activities such as wound care, specimen collection, resistive exercises, and medication prescriptions that are designed to prevent, decrease, or alleviate signs and symptoms of the individual/family/community.
3. Case Management: Activities such as coordination, advocacy, and referral that facilitate service delivery, improve communication among health and human service providers, promote assertiveness, and guide the individual/family/community toward use of appropriate resources.
4. Surveillance: Activities such as detection, measurement, critical analysis, and monitoring intended to identify the individual/family/community's status in relation to a given condition or phenomenon.
