# Supplementary material for: Characteristics of nursing interventions that improve the quality of life of people with chronic diseases. A systematic review with meta-analysis
Source: PLoS One. 2019 Jun 24;14(6):e0218903. doi: 10.1371/journal.pone.0218903 (PMC6590814; doi:10.1371/journal.pone.0218903)
Supplement: S3 File — (PDF) [file pone.0218903.s003.pdf]

The statistical analyses were conducted with the following software programs: Excel 2013, IBM SPSS 22, the Stat 12.0 commands and R-3.3.1 software along with R Studio and packages confunnel, metabias and metafor.

The intercoder agreement was estimated, resulting in a mean agreement reached for both categorical and continuous variables. Cohen's Kappa coefficient was used for the categorical values, and the Spearman-Brown correlation coefficient was used for quantitative variables.

The effect size (ES) indices were estimated by using the standard mean proposed by Hedges. The standard differences in means were calculated by finding the difference between the post and pre intervention means of each group divided by the post intervention standard deviation.

The ES index has a normal distribution with a range of values from infinite negative to infinite positive, where a value of zero represents a null effect. To interpret the value of the index, the area of research and its clinical impact must be taken into account. Nevertheless, following the classification by Cohen, an ES = 0.20 is considered small, an ES = 0.50 medium and an ES = 0.8 to infinity, large (24).

The standardized mean difference and the variance of each study were estimated with the Huedo-Medina and Johnson calculator (1). This calculation sheet uses a correction factor for small sample sizes.

The estimation of the standardized mean difference allows comparing or combining results from different types of designs. Thus, for the data extracted from the articles the estimations of the ES index were: 1/ the means or dispersion indices (standard deviations, standard error or confidence interval) pre and post intervention of the experimental group and control group, or 2/ the mean and standard deviation of the change of the experimental group and control.

To respect the principle of interdependence, if the study provided various post-intervention measurements, only one was chosen, the one that corresponded with the end of the intervention. If the end of the intervention was unknown, the one closest to 6 months was selected. Likewise, the primary studies that were suspected of containing total or partial sample dependence with another study were excluded.

In order to obtain an overall index of the magnitude of the effect, each estimate was calculated by using the inverse of its sampling variance, assuming a random model with the DerSimonian and Laird method.

Bias of the distribution of the effect sizes was analyzed through Begg's strategy and Egger's test.

The homogeneity was evaluated with the inferential Q test proposed by Cochran, and the I^2 homogeneity index with its corresponding confidence interval.

To explain the heterogeneity detected in the indices, a bivariate analysis was conducted, assuming a model of mixed effects with the restricted maximum likelihood (REML) approach. The moderating variables that had sufficient statistical power were introduced into multivariate models. Likewise, a sensitivity analysis was performed in order to detect the influence of possible extreme or atypical scores and to visualize a possible trend in the results.

1. Huedo-Medina TB, Johnson BT. Estimating the standardized mean difference effect size and its variance from different data sources: A spreadsheet. Storrs, CT, USA: Authors. 2011.
